# An Unusual Case of Spontaneous Bladder Perforation with Associated Autodialysis of the Ensuing Urinary Ascites

**DOI:** 10.1155/2011/145084

**Published:** 2011-11-16

**Authors:** A. Aber, S. A. Hyder, V. Arumuham

**Affiliations:** ^1^Barts and The London School of Medicine and Dentistry, Garrod Building, Turner Street, London E1 2AD, UK; ^2^Department of Surgery, Royal Lancaster Infirmary, Ashton Road Lancaster, Lancashire LA1 4RP, UK

## Abstract

Spontaneous rupture of the urinary bladder is a rare occurrence, and when encountered it is a diagnostic challenge. We present an unusual case of urinary bladder rupture in a patient with severe cerebral palsy who initially presented with localized abdominal pain and during admission developed generalized peritonitis caused by bladder rupture. In this case, the patient had none of risk factors associated with urinary bladder.

## 1. Background

A 47-year-old female who is aphasic with debilitating cerebral palsy, presented emergently to the Surgical Admission Unit. She is tetraplegic, with fixed flexion contractures of all four limbs, incontinent of urine and manages with pads. According to her carers, her predominant symptoms were of worsening agitation for 24 hours and localised pain to her abdomen.

**Figure 1:**
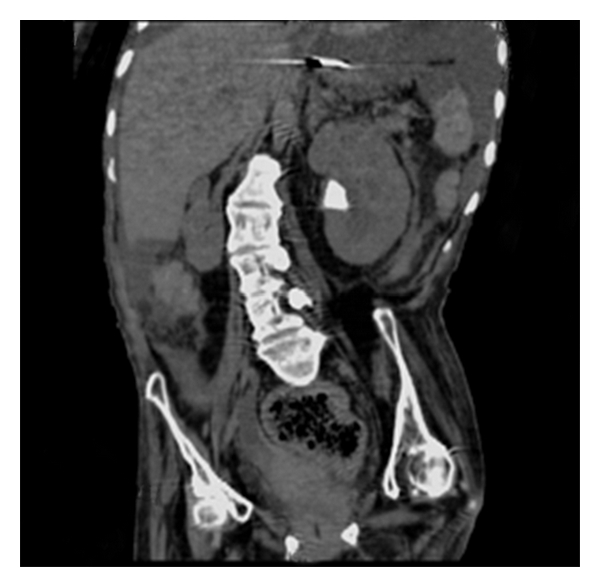
7 mm obstructing left PUJ calculus.

Examination by the posttake Consultant Surgeon on rounds described her abdomen as soft, with palpable faecal loading. However, after a stormy night morning reexamination by the same Surgeon revealed a distended, tense abdomen with evidence of intra-abdominal fluid and generalised guarding. An urgent ultrasound showed left-sided hydronephrosis with an obstructing 7 mm calculus (Figure 1) and large amounts of free peritoneal fluid.

**Figure 2:**
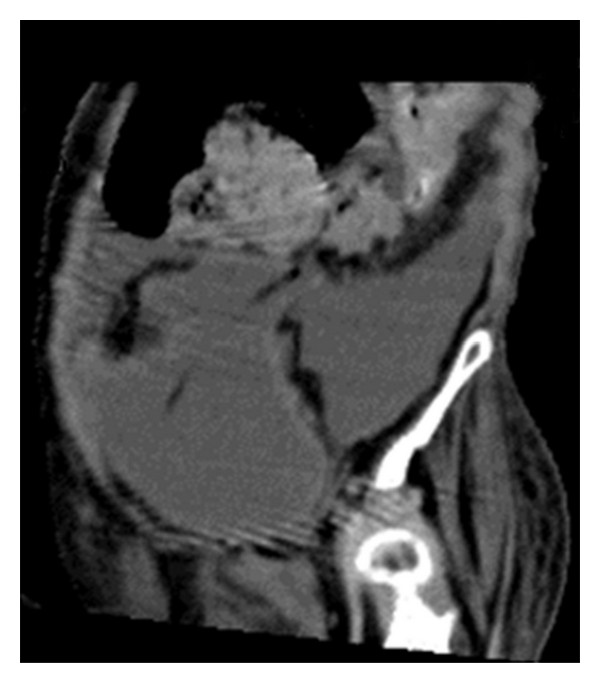
Free peritoneal fluid.

**Figure 3:**
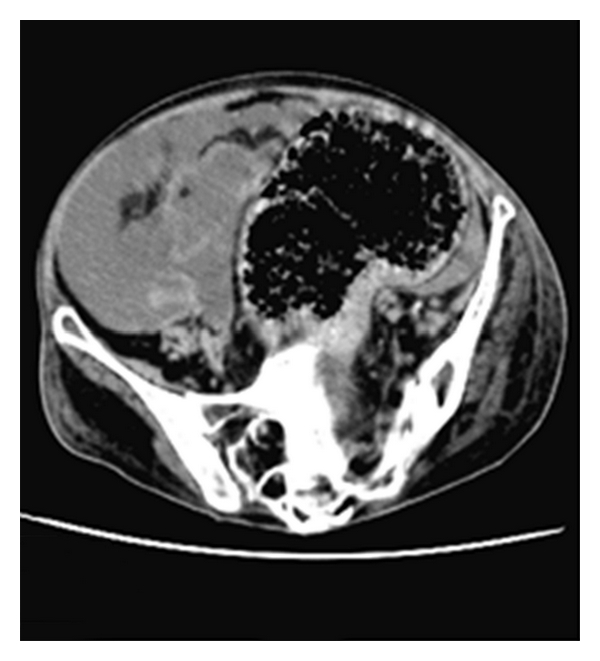
Distended sigmoid colon.

CT of the abdomen without contrast confirmed the presence of extensive free fluid (Figure 2), predominantly along the paracolic gutters and extending under the hemidiaphragms with “scalloping” of the liver. The sigmoid colon was markedly distended (Figure 3) with faeces lifting it out of the pelvis to the level of L2. On this unenhanced scan there were no radiological features suggestive of faecal peritonitis. Clear urine drained from the bladder upon easy urethral catheterisation with a documented residual of 600 mL.

Aspirate from a Diagnostic Peritoneal Tap (DPT) confirmed the presence of Nitrites/Leucocytes via Dipstix. Blood markers on admission showed an elevated CRP and sodium levels. However, repeat Urea & Electrolytes after clinical deterioration showed new onset of acute renal failure on a background of persisting hypernatraemia. In light of her worsening clinical picture she underwent an urgent exploratory Laparotomy.

Intraoperatively a distended sigmoid colon predominantly occupied the true pelvis (Figure 3), but there was no evidence of bowel ischaemia. There were intermittent areas of dilated small bowel but no obvious mechanical bowel obstruction. The bladder was grossly thickened, with a small necrotic area and three separate perforations along the posterior aspect. Many litres of colourless free fluid were present in the abdomen; however, biochemical analysis did not show the urea/creatinine concentrations within the fluid to be high enough to indicate the presence of urine. Microbiology yielded no growth.

The bladder was closed in two layers, and the wound was left with a drain in situ. A cystogram 7 days postop confirmed no leak and renal function had returned to normal. The obstructing left-sided calculus was thought to be of longterm and with stable renal function an outpatient Percutaneous Nephrolithotomy (PCNL) was planned. 

## 2. Discussion

Bladder rupture is commonly classified as intraperitoneal or extraperitoneal [1, 2]. This case demonstrates the usual triad of intra-peritoneal bladder rupture which includes abdominal pain, distension, and urinary ascites [3, 4].

Spontaneous rupture of the urinary bladder is a rare occurrence; in the available literature it is predominantly associated with risk factors such as radiotherapy for pelvic malignancies [1, 3, 4], neuropathic bladder [5], trauma, binge alcohol drinking [6], continuous bladder irrigation [7], postpartum [6, 8], bladder diverticulum [9], and in association with pelvic organ prolapse [10].

Common denominators within these risk factors are pathological bladder wall weakness and/or increased bladder pressure [11]. In the presented case we suspect that the cause of the rupture is a combination of bladder outlet obstruction (secondary to severe faecal loading) and a neuropathic bladder [11]. This is the first case to report a combination of outflow obstruction caused by faecal loading plus a neuropathic bladder as the causes of bladder rupture.

A ruptured bladder leads to urinary ascites—where free urine leaks into the peritoneal cavity. This may in itself present as lower abdominal pain, oliguria, and acute renal failure [11].

A previously normal but suddenly elevated serum urea and creatinine may be a key feature in the diagnosis of urinary ascites. The new onset of renal failure in this clinical case is based on the concept of “reverse autodialysis” of the peritoneal membrane. This is a reverse form of continuous ambulatory peritoneal dialysis, most apparent when there is a delay in presentation, whereby instead of diffusion of metabolic waste products into a dialysate. The peritoneum reabsorbs urea and creatinine from the leaked urine. This causes a subsequent rise in serum levels.

In cases where the leakage of fluid has occurred for greater than 24 hrs, the progression of reverse auto-dialysis may alter the ratio of constituents required for diagnosis. For example, in this case the DPT was positive for nitrites/leucocytes, yet the creatinine ratio was <1. It could be said that the longer the time to DPT, the less the specificity of the final result.

Unfortunately in the painless bladder and compounded by an aphasic patient this approach may not easily be applied. In the past, the gold standard for diagnosis was the cystogram [3, 7, 8, 10]; however in the acute situation with worsening physiological variables, this luxury may not always be available to the clinician. Ramcharan et al. described the importance of analysing for urinary constituents via DPT. This is objective, rapid, and sensitive in analysing intra-abdominal fluid for urine. An ascitic creatinine : serum creatinine ratio >1.0 is termed highly suggestive of an intraperitoneal urine leak [12].

The clue to the diagnosis here was the sudden onset of renal failure, which in the presence of urinary ascites has been shown to be a sensitive marker for intraperitoneal urine [4, 13]. On admission renal function was normal, corresponding with a clinically soft abdomen. However, repeated bloods after the abdomen becomes distended and guarded indicates acute renal failure.

One can postulate that DPT is only specific if done at an early stage and as time pass it becomes insufficient to use as a marker to aid in diagnosis. The advent of CT has made situations where bladder perforation is suspected more straightforward to diagnose.

With current resources and a situation where urethral trauma is not suspected a catheter instillation of contrast and subsequent CT Cystograppy can yield sensitive results [8]. In this case, certainly the vague presentation in an aphasic patient proved to be diagnostically difficult. However a DPT due to newly distended fluid-filled abdomen was thought to be more appropriate. Urinary ascites may undergo autodialysis and therefore diagnostic peritoneal tap in urinary ascites is of limited use after 24 hrs.
